# Lambs with Scrapie Susceptible Genotypes Have Higher Postnatal Survival

**DOI:** 10.1371/journal.pone.0001236

**Published:** 2007-11-28

**Authors:** Rami M. Sawalha, Susan Brotherstone, Joanne Conington, Beatriz Villanueva

**Affiliations:** 1 Scottish Agricultural College, Edinburgh, United Kingdom; 2 School of Biological Sciences, University of Edinburgh, Edinburgh, United Kingdom; Freie Universitaet Berlin, Germany

## Abstract

**Background:**

Prion protein (*PrP*) alleles associated with scrapie susceptibility persist in many sheep populations even with high frequencies despite centuries of selection against them. This suggests that scrapie susceptibility alleles have a pleiotropic effect or are associated with fitness or other traits that have been subject to selection.

**Methodology/Principal Findings:**

We genotyped all lambs in two scrapie-free Scottish Blackface sheep flocks for polymorphisms at codons 136, 154 and 171 of the *PrP* gene. We tested potential associations of the *PrP* genotype with lamb viability at birth and postnatal survival using a complementary log-log link function and a Weibull proportional hazard model, respectively. Here we show there is an association between *PrP* genotype, as defined by polymorphisms at codons 154 ad 171, and postnatal lamb survival in the absence of scrapie. Sheep carrying the wild-type ARQ allele have higher postnatal survival rates than sheep carrying the more scrapie-resistant alleles (ARR or AHQ).

**Conclusion:**

The *PrP* genotypes associated with higher susceptibility to scrapie are associated with improved postnatal survival in the absence of the disease. This association helps to explain the existence, and in many instances the high frequency, of the ARQ allele in sheep populations.

## Introduction

The polymorphisms at codons 136, 154 and 171 of the prion protein (*PrP*) gene have been shown to be associated with susceptibility to scrapie and to have a major effect on the survival of infected animals [1], [2]. Additionally, in scrapie-affected flocks, animals with susceptible *PrP* genotypes have a higher incidence of death from unknown causes than animals with resistant genotypes [3], [4]. Thus, the persistence of *PrP* alleles associated with scrapie susceptibility suggests that the gene has a pleiotropic effect or is linked to other genes on ovine chromosome 13 that affect fitness, health or performance in the absence of scrapie. Examples of such genes include interleukin 2 receptor alpha, a gene involved in antibody production [5]; the gene coding for centromere protein B, an antibody binding protein [6], [7]; and the matrix metalloproteinase 9 gene [8] which may have a role in tumor invasion and metastasis [9]. However, genetic-based scrapie eradication programs ignore the possibility of association of *PrP* gene with other traits and rely on polymorphisms at three codons of the *PrP* gene through selecting in favor of the alleles known to confer the highest resistance (e.g. ARR) and against those associated with the highest susceptibility (e.g. VRQ).

Several recently-published studies have reported no clear associations of polymorphisms in the *PrP* gene with growth and reproductive traits [10]–[15] but associations with survival have not been investigated. Nevertheless, there is a view amongst many sheep breeders in the UK that susceptible sheep outperform resistant sheep [16] and that the wild-type allele (ARQ) [17] is associated with superior survival under harsh environmental conditions. This view is supported by the fact that a higher frequency of this allele (ARQ) can be observed in hill sheep breeds, mostly raised under harsher environments, compared to breeds raised under less environmentally challenging conditions [18]. The existence of the ARQ allele (known to be associated with high to moderate susceptibility to scrapie) in all sheep breeds and its high frequency in many hardy breeds, despite the fact that scrapie has been present for over 250 years, suggests that this allele has selective advantage for fitness [17].

## Materials and Methods

All lambs in two scrapie-free (i.e. no reported clinical cases of scrapie) Scottish Blackface flocks of the Scottish Agricultural College were genotyped. One of the flocks is located in the Pentland Hills in Midlothian and the other flock is located in West Perthshire, Scotland. The animals in the two flocks are genetically connected through the use of 2 rams to inseminate 40 ewes in each flock every year. The animals were managed in a similar way to commercial hill sheep but were comprehensively recorded [19]. We considered polymorphisms at codons 136, 154 and 171 of the *PrP* gene and found four *PrP* alleles (ARR, ARQ, AHQ and VRQ). We obtained *PrP* genotypes for lambs born from 1999 to 2004 from blood samples taken around weaning age (120 days). Genotypes for lambs born from 2002 to 2004 that died before weaning were obtained from ear tissue samples. Genotypes were obtained utilizing proprietary SNP technology. All procedures involving animals were in accordance with the guidelines of the animal ethics committee at Scottish Agricultural College and were carried out under the United Kingdom Home Office license, following the regulations of the Animals Act 1986.

We tested potential associations of the *PrP* genotype with four lamb survival traits: viability at birth (VB), survival from 1 d to 14 d (S1-14), survival from 15 d to 120 d (S15-120) and survival from 121 d to 180 d (S121-180). There were 3,955 records for VB, 3,743 records for S1-14, 3,673 records for S15-120 and 6,777 records for S121-180 (Table S1). Viability at birth was defined as a binary trait and lambs were considered viable if they were born alive and survived for 24 h after birth. The VB data were analyzed using a complementary log-log link function with the statistical software ASREML [20]. For the postnatal periods, the number of days before death or until the end of the period (censored) were recorded. Postnatal survival traits were analyzed using a Weibull proportional hazard model with the Survival Kit [21]. The models included the biologically-sensible fixed effects (sex, type of birth or rearing, year of birth, age of dam and flock) when statistically significant (P<0.05) as described elsewhere [22]. The models also included random sire effect to account for polygenic effects.

The effect of the *PrP* genotype was estimated by including it as a fixed factor in the model. We estimated associations between survival and alleles of the *PrP* gene by classifying the *PrP* genotypes in 5 different ways. In the first four analyses, animals were classified according to the number of copies of each of the *PrP* alleles they carried. For example, analysis I was based on the number of copies of the ARR allele and there were three levels for the *PrP* genotype (ARR homozygous, ARR heterozygous and ARR non-carriers). For alleles AHQ and VRQ, only two levels were used (excluding the homozygous genotypes) as their frequency was either too small (AHQ/AHQ, 0.68) or zero (VRQ/VRQ). We based the last *PrP* genotypic classification (analysis V) on the five most common genotypes, namely, ARR/ARR, ARR/ARQ, ARR/AHQ, ARQ/ARQ and ARQ/AHQ. The hazard ratios for ARR/ARR, ARR/AHQ, ARQ/ARQ and ARQ/AHQ genotypes were compared relative to ARR/ARQ genotype as it was found to generally have the lowest hazard rate compared with the other genotypes (see results section). Statistical tests were performed using the Bonferroni-corrected likelihood-ratio test.

## Results

### PrP allele and genotypic frequency

The population consisting of animals from the two flocks did not significantly deviate from Hardy-Weinberg equilibrium and had all expected combinations of *PrP* alleles except the VRQ/VRQ genotype. The ARQ was the most frequent allele (60.1%) followed by ARR (31.2%), AHQ (7.7%) and VRQ (1.0%) (Figure S1).

### Association of PrP genotype and lamb survival

The *PrP* alleles showed no significant association with viability at birth but influenced the hazard ratio (i.e. relative likelihood of death) during all postnatal periods (Table 1). The largest effects of the *PrP* alleles were associated with the presence or absence of ARR and ARQ and, to a lesser extent, with the AHQ. These three alleles arise from polymorphisms at two codons (154 and 171) of the *PrP* gene. Generally, the presence of the ARQ allele was associated with a lower hazard ratio while the presence of the ARR allele or the AHQ allele was mostly associated with an increased hazard ratio. Specifically, we found that the postnatal hazard ratio was significantly influenced by the presence or the absence of ARR and ARQ alleles for S1-14 and S121-180. During these periods, the hazard ratio was more than two times higher for ARR/ARR lambs than for ARR heterozygous lambs (Table 1). Comparatively, ARQ heterozygous lambs showed two to three times lower hazard ratio than ARQ non-carriers during the same periods (S1-14 and S121-180). ARQ homozygous lambs were also at a lower postnatal hazard rate than lambs without the ARQ allele but the ratios were not statistically significant. The postnatal hazard ratio was also significantly affected by the AHQ allele with carriers at two times greater risk than non-carriers for the S15-120 period. The postnatal hazard ratios were not affected by the presence or absence of the VRQ allele (Table 1).

**Table 1:** Hazard ratios (and s.e.) between different *PrP* genotypes

Ratio	Survival period^a^		
	S1-14 (Ratio)	S15-120 (Ratio)	S121-180 (Ratio)
**Analysis I** b,c			
ARR/ARR to ARR/xxx	2.67 (0.74)*	0.81 (0.25)	2.34 (0.58)*
ARR/ARR to xxx/xxx	2.94 (0.83)*	0.93 (0.30)	1.70 (0.41)
ARR/xxx to xxx/xxx	1.10 (0.28)	1.16 (0.25)	0.73 (0.14)
**Analysis II**			
ARQ/ARQ to ARQ/xxx	1.24 (0.33)	0.72 (0.18)	1.29 (0.26)
ARQ/ARQ to xxx/xxx	0.44 (0.12)	0.46 (0.13)	0.62 (0.14)
ARQ/xxx to xxx/xxx	0.36 (0.09)*	0.64 (0.16)	0.48 (0.11)*
**Analysis III**			
AHQ/xxx to xxx/xxx	0.82 (0.26)	2.64 (0.60)*	1.58 (0.34)
**Analysis IV**			
VRQ/xxx to xxx/xxx	0.99 (0.44)	1.61 (0.62)	1.00 (1.00)

a S1-14: survival from 1 d to 14 d; S15-120: survival from 15 d to 120 d; S121-180: survival from 121 d to 180 d.

b Analysis I: genotypes were classified as ARR/ARR, ARR/xxx and xxx/xxx where xxx is any allele other than ARR; Analysis II: genotypes were classified as ARQ/ARQ, ARQ/xxx and xxx/xxx where xxx is any allele other than ARQ; Analysis III: genotypes were classified as AHQ/xxx and xxx/xxx where xxx is any allele other than AHQ; Analysis IV: genotypes were classified as VRQ/xxx and xxx/xxx where xxx is any allele other than VRQ.

c Ratios with “*” are significantly different from 1 (P<0.05) after adjustment for multiple tests using Bonferroni correction.

**Figure 1:**
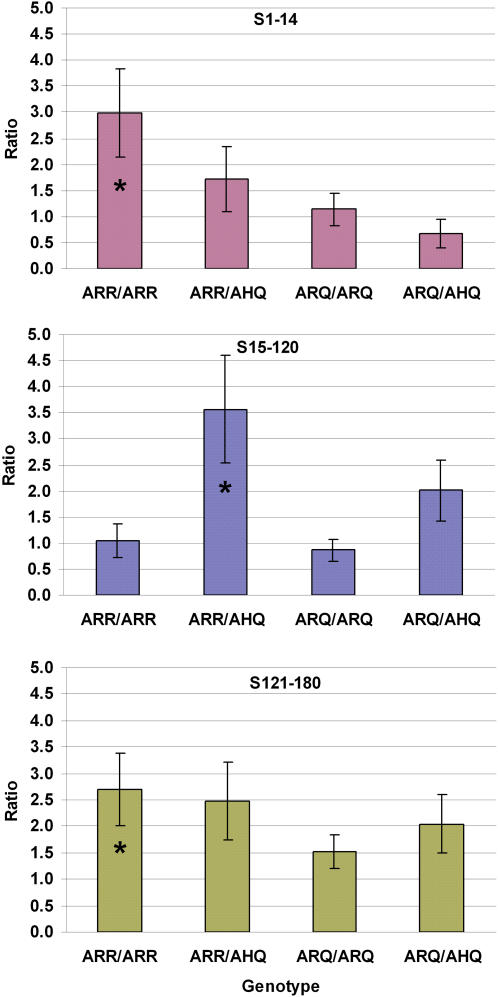
Hazard Ratios of PrP Genotypes. Comparison of hazard ratios and their s.e. for lambs with different *PrP* genotypes during three postnatal periods. S1-14: survival from 1 d to 14 d; S15-120: survival from 15 d to 120 d; S121-180: survival from 121 d to 180 d. Genotypes ARR/ARR, ARR/AHQ, ARQ/ARQ and ARQ/AHQ were compared relative to ARR/ARQ genotype. Hazard ratios with “*” are significantly different from 1 (P<0.05) after adjustment for multiple tests using Bonferroni correction.

To find the specific genotypes associated with lamb survival, we compared the hazard ratios of ARR/ARR, ARR/AHQ, ARQ/ARQ and ARQ/AHQ genotypes relative to ARR/ARQ genotype (analysis V). With this analysis, we found that the ARR/ARQ genotype was associated with a lower hazard of postnatal mortality than the ARR/ARR and ARR/AHQ genotypes. Specifically, ARR/ARR genotype had a significantly (P<0.05) higher hazard ratio than ARR/ARQ genotype during S1-14 and S121-180 periods. Similarly, lambs with the ARR/AHQ genotype had significantly higher hazard ratio than those with the ARR/ARQ genotype during S15-120 period (Figure 1).

### Test of dominance effect of PrP genotypes and lamb survival

**Figure 2:**
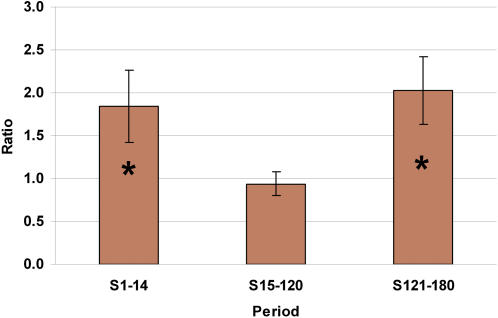
Test of Dominance between ARR and ARQ Alleles. The ratios are for testing dominance interaction between ARR and ARQ alleles by comparing the hazard ratios between the geometric mean of the homozygous genotypes (ARR/ARR and ARQ/ARQ) relative to the ARR/ARQ genotype during three postnatal periods. S1-14: survival from 1 d to 14 d; S15-120: survival from 15 d to 120 d; S121-180: survival from 121 d to 180 d. Periods with “*” indicate significant (P<0.05) dominance effect.

Based on the estimates of hazard ratios involving the ARR and ARQ alleles, we conducted a test to investigate possible dominance interaction between these alleles. In a subset of the data with only ARR/ARR, ARR/ARQ and ARQ/ARQ genotypes, we compared the hazard rate of the heterozygous to the geometric mean of the two homozygous genotypes. There was significant evidence of a complete dominance effect between the ARR and ARQ alleles (Figure 2). Homozygous lambs for the ARR and ARQ alleles were at about two fold greater hazard rate than heterozygous lambs for the S1-14 and S121-180 periods. However, we did not find significant evidence of overdominance when the hazard rate associated with ARR/ARQ genotype was compared with that of ARQ/ARQ (Figure 1).

## Discussion

Lamb survival traits are complex traits with several, possibly interacting, factors affecting them. The models used to analyze the data accounted for both random genetic and fixed environmental effects. The estimate of the genetic variance was significant for all traits along with fixed effects such as sex, age of dam, type of birth or rearing, year of birth and flock [22]. The size and structure of the dataset allows us to confidently partition variation in each survival trait into environmental, *PrP* and non-*PrP* polygenic genetic effects, minimizing the chance of spurious effects or failure to detect a real effect. Because of the significant effects of both flock and *PrP* genotype, we tested if survival was differently affected in different flocks by *PrP* genotype. There was no evidence of genotype by environment interaction as the differences in the hazard rate were not significantly different between the two flocks (P>0.05).

Lamb survival is of critical economic and welfare importance to sheep enterprises, where an average of 10% and up to 40% of the total lamb crop can be lost during the neonatal period under temperate climates such as that in the UK [23], [24]. The increased hazard of mortality for some *PrP* genotypes can be quantified by comparing the rate of mortality for different genotypes when defining mortality within periods as a binary trait. When doing so, the estimated lamb loss from 1 d to 180 d due to higher postnatal mortality for the ARR/ARR genotype compared with the ARR/ARQ genotype was 2.20% (Table S2). This figure was obtained as the sum of the difference in the mortality rate over the considered periods after accounting for the fact that animals that died in the first period (S1-120) should be excluded from the calculation of mortality rate in the later period (S121-180). In other words, out of 1000 lambs born alive, about 22 more lambs are expected to die if they are of the ARR/ARR genotype than if they are of the ARR/ARQ genotype. Based on this, the average effect of gene substitution (ARR with ARQ) in the population studied is a reduction in the mortality rate of 0.34%. However, the VRQ allele showed no significant association with lamb survival which is notable: the allele with the biggest impact on scrapie susceptibility has no effect on survival under scrapie-free conditions. This result should be interpreted with caution due to the low frequency of the VRQ allele and therefore the VRQ carriers (67 to 136 depending on the trait).

There are no results in the literature on the possible effects of the *PrP* gene on lamb survival in scrapie-free flocks. However, several studies have been published recently investigating associations of the *PrP* gene with performance traits in sheep. Most of the lamb and ewe performance traits studied were found to be not affected by *PrP* genotype. However, in a previous study of Scottish Blackface lambs we found a significant association of the ARQ allele with birth weight [15]. In order to investigate whether the differences in survival we found between different *PrP* genotypes are explainable by differences in birth weight we fitted birth weight as a covariate in the survival model. We found that birth weight has a high significant effect (P<0.001) on all survival traits except in the later period (S121-180). However, adjusting survival traits for birth weight did not result in major differences in the results of association of *PrP* genotypes with postnatal lamb survival (Figure S2). Therefore, the variation of body weight at birth does not explain the observed associations between survival and *PrP* genotypes.

In scrapie-affected flocks, survival of animals without clinical scrapie has been found to be significantly less for animals with the susceptible *PrP* genotypes than for animals with the resistant ones [25]. The higher mortality rate for animals with the susceptible genotypes could be attributed to preclinical scrapie or to possible deleterious effects of some *PrP* alleles in the presence of scrapie in the flock. However, the elevated risk of mortality for some *PrP* genotypes found in our study could not be related to preclinical scrapie, as the flocks are known to be scrapie free. Also, the lambs with known resistant genotypes (ARR/ARR and ARR/AHQ) were at higher risk of postnatal mortality than other more scrapie-susceptible genotypes (eg ARQ/ARQ) and the survival periods considered were at young ages (not more than 6 months). Likewise, we did not find evidence of mortality due to undiagnosed scrapie cases in the two flocks, as there were no differences in the number of observed and expected ARR/VRQ animals (from allele frequency). In other words, if there had been a scrapie outbreak, the frequency of the most susceptible allele (VRQ) would have become too low to account for the observed frequency of the resistant ARR/VRQ genotype (scrapie signature) [2]. Therefore, the equilibrium between the expected and observed numbers for ARR/VRQ genotype indicates that there has not been a scrapie outbreak in the flocks studied.

Studies on the physiological role of the *PrP* gene (mostly in knockout mice) have found that the gene may be necessary for normal functioning of several nervous system processes, circadian rhythm, survival of some nervous cells and may be related to oxidative stress and post-hypoxia neuronal responses [26]–[29]. A malfunction in some of these vital processes might have contributed to the higher risk of lamb mortality observed for some *PrP* genotypes.

Our results strongly suggest a higher viability of the animals with the scrapie susceptible ARQ allele in the absence of scrapie. This allele is considered as the wild (ancestral) allele as all other alleles are only different from it by a single nucleotide substitution and it is present in all sheep breeds with noticeably high frequency in some genetically isolated breeds such as Soay and Icelandic sheep [17]. The selective superiority of the ARQ allele in the absence of scrapie helps to explain its persistence in sheep populations. Our results support the findings that *PrP* gene is under balancing selection as determined using molecular evolution techniques comparing the ratios of synonymous (amino acid non-changing) and nonsynonymous polymorphisms [30]. A similar phenomenon is known for *Plasmodium falciparum* malaria in that susceptibility to infection is controlled by the host genotype and susceptibility alleles have selective superiority in the absence of infection.

The selective advantage of animals with the ARQ allele in the absence of scrapie is not offset by its association with higher susceptibility to scrapie during disease outbreaks. We compared the selective forces of ARR/ARR, ARR/ARQ and ARQ/ARQ genotypes due to scrapie mortality and lifetime breeding success in the presence of scrapie [25], [31], and found it to be too small to balance the selective advantage of the ARQ allele on postnatal survival in the absence of scrapie which can result in much faster changes of the ARQ frequency in the opposite direction. The *PrP* allelic and genotypic frequencies in the studied flocks were in general agreement with those estimated in the UK Scottish Blackface population [18]. These results prove that the overall ARQ allele frequency is not going to decrease and leads to considering scrapie as an endemic disease that is not going to disappear due to natural selection through genetic-based scrapie susceptibility alone. The selective advantage of the ARQ allele on postnatal survival compared to ARR may also hinder the current scrapie eradication programs relying on selective breeding based on *PrP* genotyping.

## Supporting Information

Table S1 (0.03 MB DOC)

Table S2 (0.03 MB DOC)

Figure S1 (0.03 MB DOC)

Figure S2 (0.03 MB DOC)
