# Supplementary material for: Lambs with Scrapie Susceptible Genotypes Have Higher Postnatal Survival
Source: PLoS One. 2007 Nov 28;2(11):e1236. doi: 10.1371/journal.pone.0001236 (PMC2077931; doi:10.1371/journal.pone.0001236)
Supplement: Table S1 — (0.03 MB DOC) [file pone.0001236.s001.doc]

| Table S1. **Number of records, mortality rate and mean age at death^1^** | | | |
| --- | --- | --- | --- |
| Trait | Number | Mortality rate (%) | Mean age of mortality (d) |
| Viability at birth | 3,955 | 5.69 | NA |
| Survival from 1 d to 14 d | 3,743 | 1.23 | 4.41 |
| Survival from 15d to 120 d | 3,673 | 1.54 | 60.3 |
| Survival from 121 d to 180 d | 6,777 | 1.59 | 148 |
| ^1^ Data were available for lambs born in 1999 through 2004 for survival from 121 d to 180 d and from 2002 through 2004 for the other traits. | | | |
