# Supplementary material for: Lambs with Scrapie Susceptible Genotypes Have Higher Postnatal Survival
Source: PLoS One. 2007 Nov 28;2(11):e1236. doi: 10.1371/journal.pone.0001236 (PMC2077931; doi:10.1371/journal.pone.0001236)
Supplement: Table S2 — (0.03 MB DOC) [file pone.0001236.s002.doc]

| **Table S2.** Mortality rate (%) (and s.e.) by *PrP* genotype^1^ | | |
| --- | --- | --- |
|  | Survival period^2^ | |
| Genotype | S1-120 | S121-180 |
| ARR/ARR | 3.73 (1.31) | 1.26 (0.53) |
| ARR/ARQ | 2.25 (0.67) | 0.49 (0.18) |
| ARQ/ARQ | 2.65 (0.78) | 0.74 (0.26) |
| ^1^ The analysis was done using binary defined survival traits.  ^2^ S1-120: survival from 1 d to 120 d; S121-180: survival from 121 d to 180 d. | | |
