# Supplementary material for: Lambs with Scrapie Susceptible Genotypes Have Higher Postnatal Survival
Source: PLoS One. 2007 Nov 28;2(11):e1236. doi: 10.1371/journal.pone.0001236 (PMC2077931; doi:10.1371/journal.pone.0001236)
Supplement: Figure S1 — (0.03 MB DOC) [file pone.0001236.s003.doc]

***Figure S1. Allele and Genotype Frequencies of PrP Gene***

|  |
| --- |
|  |
