# Supplementary material for: Lambs with Scrapie Susceptible Genotypes Have Higher Postnatal Survival
Source: PLoS One. 2007 Nov 28;2(11):e1236. doi: 10.1371/journal.pone.0001236 (PMC2077931; doi:10.1371/journal.pone.0001236)
Supplement: Figure S2 — (0.03 MB DOC) [file pone.0001236.s004.doc]

***Figure S2. Hazard Ratios of PrP Genotypes with Birth Weight Adjustment***

|  | Comparison of hazard ratios and their s.e. for lambs with different *PrP* genotypes during three postnatal periods with adjustment for birth weight. S1-14: survival from 1 d to 14 d; S15-120: survival from 15 d to 120 d; S121-180: survival from 121 d to 180 d. Genotypes ARR/ARR, ARR/AHQ, ARQ/ARQ and ARQ/AHQ were compared relative to ARR/ARQ genotype. Hazard ratios with “*” are significantly different from 1 (P < 0.05) after adjustment for multiple tests using Bonferroni correction. |
| --- | --- |
|  |
|  |
